# Supplementary material for: Online harassment of Japanese celebrities and influencers
Source: Front Psychol. 2024 Apr 15;15:1386146. doi: 10.3389/fpsyg.2024.1386146 (PMC11057462; doi:10.3389/fpsyg.2024.1386146)
Supplement: Supplementary file 1 [file Data_Sheet_1.PDF]

Supplementary Information

Harassment type	Private-Collective	Private-Individual	Public-Collective	Public-Individual
Appearance abuse	0.08	0.07	0.15	0.15
Ability abuse	0.14	0.12	0.25	0.24
Personality abuse	0.13	0.11	0.26	0.22
Obscene massages	0.07	0.06	0.09	0.09
Intimidation	0.06	0.05	0.05	0.08
Requesting a sexual relationship	0.08	0.08	0.06	0.08

Table S1. Direct harassment frequencies of offender types and methods

Harassment type	Frequency
My photos and videos were uploaded without authorization	0.19
My sexually suggestive photos and videos were uploaded	0.03
My ungainly photographs and videos were uploaded	0.10
Photoshopped my photos and videos were uploaded	0.04
My personal information was disclosed	0.09
I was asked for help in situations that were difficult to respond for them to	0.21
My disinformation was spread on social media	0.31
My disinformation was published on curation and news sites	0.16

Table S2. Indirect harassment frequencies

Emotional damage	Disagree	Slightly disagree	Slightly agree	Agree
I wanted to remove my account	0.55	0.12	0.21	0.12
I didn't want to use the Internet	0.41	0.07	0.36	0.17
I felt less motivated to go out and interact socially	0.6	0.12	0.17	0.11
I had a sinking feeling for several days	0.34	0.14	0.26	0.25
I wanted to be truant or leave my job	0.59	0.09	0.20	0.13
I became suicidally depressed	0.75	0.08	0.09	0.07
I indulged in intemperateness and substance abuse	0.68	0.16	0.09	0.07
I engaged in self-harm and wrecked some things	0.92	0.05	0.02	0.01

Table S3. Emotional damages

Action	Frequency
I avoided and consigned weblog/social media posts to my staff	0.07
I restricted comments and replies on my weblog/social media,	0.59
I blocked/muted harasser accounts on platforms	0.74
I reported harassers to platforms	0.5
I stopped updating my weblog/social media	0.06
I contacted inquiry counters of the platforms	0.22
I talked family/friends/business friends	0.57
I reported my victimization to my talent agency	0.15
I went to mental health counselors	0.06
I discussed with my legal consultants	0.12
I spoke to the police	0.09

Table S4. Action against online harassment

Offline harassment	Online harassment victim	Others
I received persistent requests for dating and companionship	0.14	0.02
I was stalked, ambushed, and intruded on	0.11	0.02
I was followed by a stranger	0.10	0.02
I was informed that I was being monitored	0.08	0.00
A GPS device was attached or tracking applications were installed without any prior consent	0.01	0.00
I uninterruptedly received calls, faxes, and e-mails	0.11	0.00
I received unwanted letters and/or presents	0.09	0.01
I was sent body fluid and/or excrement	0.00	0.00
I was bugged, and/or I received a present with a bugging device	0.01	0.00

Table S5. Association between offline stalkings and online harassment

ネットハラスメント実態調査

アンケート回答のお願い

* 1. このアンケートは、各メディアやインターネットで活動されている方に対して、(1)ネット上での嫌がらせ経験、(2)オフラインでのつきまとい等の被害経験についてお尋ねするものです。ご本人の体験をもとに回答してください。

このアンケートでは、安心して、読者との交流を行い、ご自身がお伝えしたい内容をそのまま投稿できる環境を実現させることを目的とし、人権が守られるネット環境へと改善するための啓発や政策立案、学術研究に活用させていただきたく考えております。

それ以外の目的以外に利用することはございません。

本調査は株式会社サイバーエージェントと一般社団法人・社会調査支援機構チキラボが共同で実施するものです。アンケートの回答は、サイバーエージェントおよびチキラボが安全に管理いたします。サイバーエージェントとチキラボ以外には個別の回答データおよび回答者を推定できる形式のデータを共有することはありません。

お名前や電話番号など、個人を直接特定できる情報は伺いません。また、回答からご本人を推定される懸念がある質問（年齢・性別など）は、「回答しない」ことを選択していただくこともできます。回答を集計したものを調査結果として公表することがありますが、個別の内容を公表することはいたしません。また、調査結果公表の際には公表する情報から回答者が類推されることがないように万全の注意を払います。

上記をご了解の上、アンケートにご協力頂ける場合は、「同意する」を選んで先に進んでください。

◯ 同意する　　◯ 同意しない

* 2. 15歳以下の方は保護者の方も本アンケートの回答に同意していることを確認してください。

◯ 16歳以上

◯ 15歳以下で保護者の同意がある

◯ 15歳以下で保護者の同意がない（アンケートを終了します）

Fig. S1. Screenshot of the survey of this research. See Fig. S2 for the English version.

Online harassment survey

Request your response to this survey

* 1. This questionnaire asks those online about (1) their experiences of online harassment and (2) their experiences of being stalked or otherwise victimized offline. Please answer based on your own experiences.

This questionnaire aims to create an environment in which you can communicate with readers safely and comfortably and post your information as you wish to convey it. We would like to use this information for awareness-raising, policy-making, and academic research to improve the Internet environment to protect human rights.

We will not use the information for any other purposes.

This survey is conducted jointly by CyberAgent, Inc. and Chiki lab, a general incorporated association and social research support organization. CyberAgent and Chiki lab will securely manage responses to the survey. Individual response data and data in a form that allows us to infer respondents will not be shared with anyone other than CyberAgent and Chiki lab.

We do not ask for any information that can directly identify you, such as your name or phone number. You may also choose not to answer any questions (e.g., age, gender, etc.) for which there is a concern that your identity may be inferred from your responses. We may release the survey results by tabulating the answers, but we will not release individual details. When publishing the survey results, we will take every precaution to ensure that the respondents cannot be identified by analogy from the information to be published.

If you agree to the above and are willing to cooperate with the survey, please select "I agree" to proceed.

○ I agree ○ I disagree

* 2. If you are 15 or younger, please ensure your parent or guardian also agrees to complete this questionnaire.

○ 16 years old and older

○ 15 years old or younger and have parental consent

○ 15 years old or younger and do not have parental consent (Exit the survey)

Fig. S2. English version of the screenshot of the survey of this research. We did not use this version because all survey were conducted in Japanese (Fig. S1).
